# Intravascular large B-cell lymphoma with respiratory failure diagnosed by random skin biopsy: A case report

**DOI:** 10.1016/j.rmcr.2024.102115

**Published:** 2024-09-15

**Authors:** Haruka Fujioka, Kei Nakashima, Yuka Uesugi, Miki Nyui, Naoki Inoshima, Jun Hayashi, Michinori Yoshimi, Miho Katsumata, Masahiro Masuzawa, Hayato Usami, Akane Ito, Rikako Tabata, Kosei Matsue

**Affiliations:** a Department of Pulmonology, Kameda Medical Center, Chiba, Japan; b Department of Hematology and Oncology, Kameda Medical Center, Chiba, Japan; c Division of Hematology, Department of Medicine, Showa University School of Medicine, Tokyo, Japan; d Department of Pathology, Kameda Medical Center, Chiba, Japan; e Department of Diabetes and Endocrinology, Kameda Medical Center, Chiba, Japan; f Department of Diabetes and Endocrinology, Nara Medical University, Nara, Japan; g Department of General Medicine, Kameda Medical Center, Chiba, Japan

**Keywords:** Intravascular large B-Cell lymphoma, Respiratory failure, Random skin biopsy

## Abstract

Intravascular large B-cell lymphoma (IVLBCL) typically involves nonspecific symptoms that complicate diagnosis. This report discusses the case of a 70-year-old man, who presented with dyspnea, fatigue, and weight loss that evolved into severe respiratory failure, diagnosed with IVLBCL via random skin biopsy. The initial improvement in respiratory symptoms was followed by coronavirus disease. Response to steroid therapy and elevated lactate dehydrogenase levels suggested IVLBCL, confirmed by a random skin biopsy. The combination chemotherapy of rituximab, cyclophosphamide, vincristine, doxorubicin, and prednisolone improved the respiratory condition. This case highlights the diagnostic challenges associated with IVLBCL and the crucial role of random skin biopsy.

## Highlights

• IVLBCL is characterized by tumor cells proliferating within small blood vessels.
• IVLBCL can be hard to diagnose due to nonspecific imaging findings and symptoms.
• This case was eventually diagnosed with IVLBCL through a random skin biopsy.
• It's vital to consider IVLBCL with unexplained severe respiratory failure.

## 1 Introduction

Intravascular large B-cell lymphoma (IVLBCL) is an aggressive hematologic malignancy characterized by the proliferation of tumor cells within small vessels such as capillaries [1]. IVLBCL lesions are frequently observed in the brain and skin, with 54 % of the patients who presented with neurological symptoms and 32 % exhibiting skin rashes [2]. The symptoms are nonspecific, with many patients presenting with fever, general fatigue, weight loss, and night sweats [3]. Although the treatment response is not unfavorable, the difficulty in considering the disease due to nonspecific symptoms and the time it takes to diagnose IVLBCL lead to delayed treatment initiation, resulting in poor prognosis [4]. Reportedly, approximately 20 % of the patients with IVLBCL are diagnosed during pathological autopsy [3].

Among the cases of pathological autopsies of IVLBCL, 60 % have been reported to involve lung lesions [5]. Therefore, pulmonary involvement is common in patients with IVLBCL. However, cases in which pulmonary lesions precede the involvement of other organs are rare [6]. Moreover, pulmonary lesions in IVLBCL do not exhibit specific symptoms but lead to respiratory distress, hypoxemia, and severe pulmonary hypertension that progress rapidly [7]. Pulmonary lesions in IVLBCL rarely form lymphadenopathy or tumor shadows on imaging studies [8] and, typically, manifest as interstitial shadows, including ground-glass opacities, that are occasionally apparently normal [9]. Owing to nonspecific findings on physical examinations and imaging studies, the diagnosis and initiation of treatment may be delayed, leading to unfavorable prognosis by the time the diagnosis is confirmed [10].

Herein, we describe the case of a patient who presented with exertional dyspnea and was diagnosed with pulmonary IVLBCL based on a random skin biopsy, and subsequently underwent chemotherapy, resulting in an improvement in respiratory status.

## 2 Case report

The patient was a 70-year-old man with no relevant medical history. Four months before visiting our hospital, he began experiencing generalized fatigue and lost 5 kg of weight. He visited a nearby hospital and underwent upper and lower endoscopic examinations that revealed no abnormalities. Contrast-enhanced computed tomography (CT) of the cervical, thoracic, abdominal, and pelvic regions revealed mild mediastinal lymphadenopathy; however, no other abnormalities were noted. Thus, the patient was referred to our hospital for further evaluation. He worked at the front office of an accommodation facility and lived with his wife. He had a history of smoking 20 cigarettes per day for 30 years but had quit smoking 20 years ago. He did not consume alcoholic drinks. Owing to the presence of generalized fatigue at the time of the visit and low cortisol levels (4.9 μg/dL) as well as low thyroid stimulating hormone (TSH) level (0.054 μU/mL), endocrine dysfunction was suspected by an endocrinologist. In the cranial contrast-enhanced magnetic resonance imaging examination, the pituitary gland appeared slightly enlarged, and a decreased enhancement effect was observed in the anterior portion of the pituitary gland. No other significant abnormal findings were identified. The patient was scheduled for further evaluation but became immobile and was urgently transported to the hospital.

**Fig. 1:**
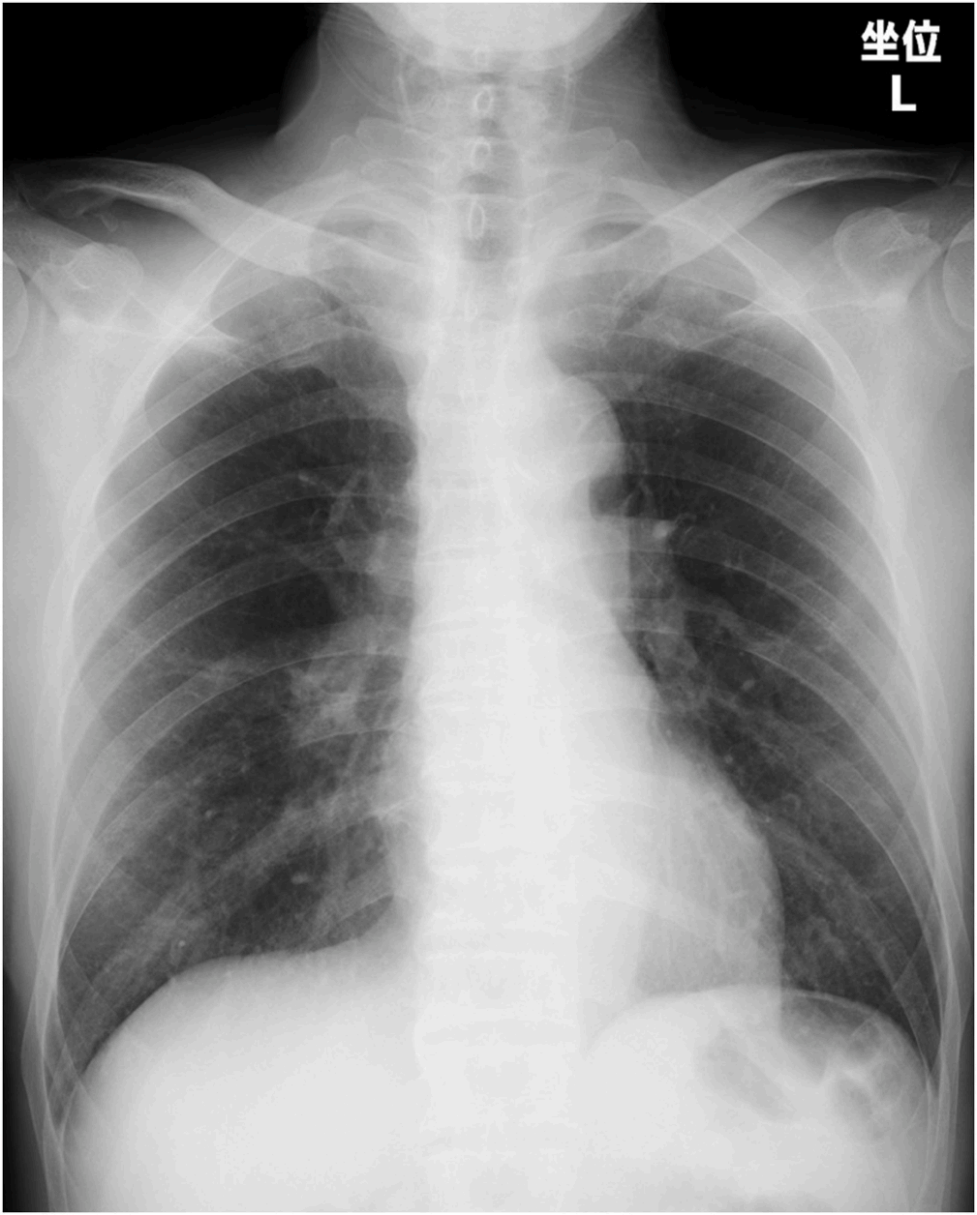
Chest radiograph of the initial visit showed no significant abnormalities.

**Fig. 2:**
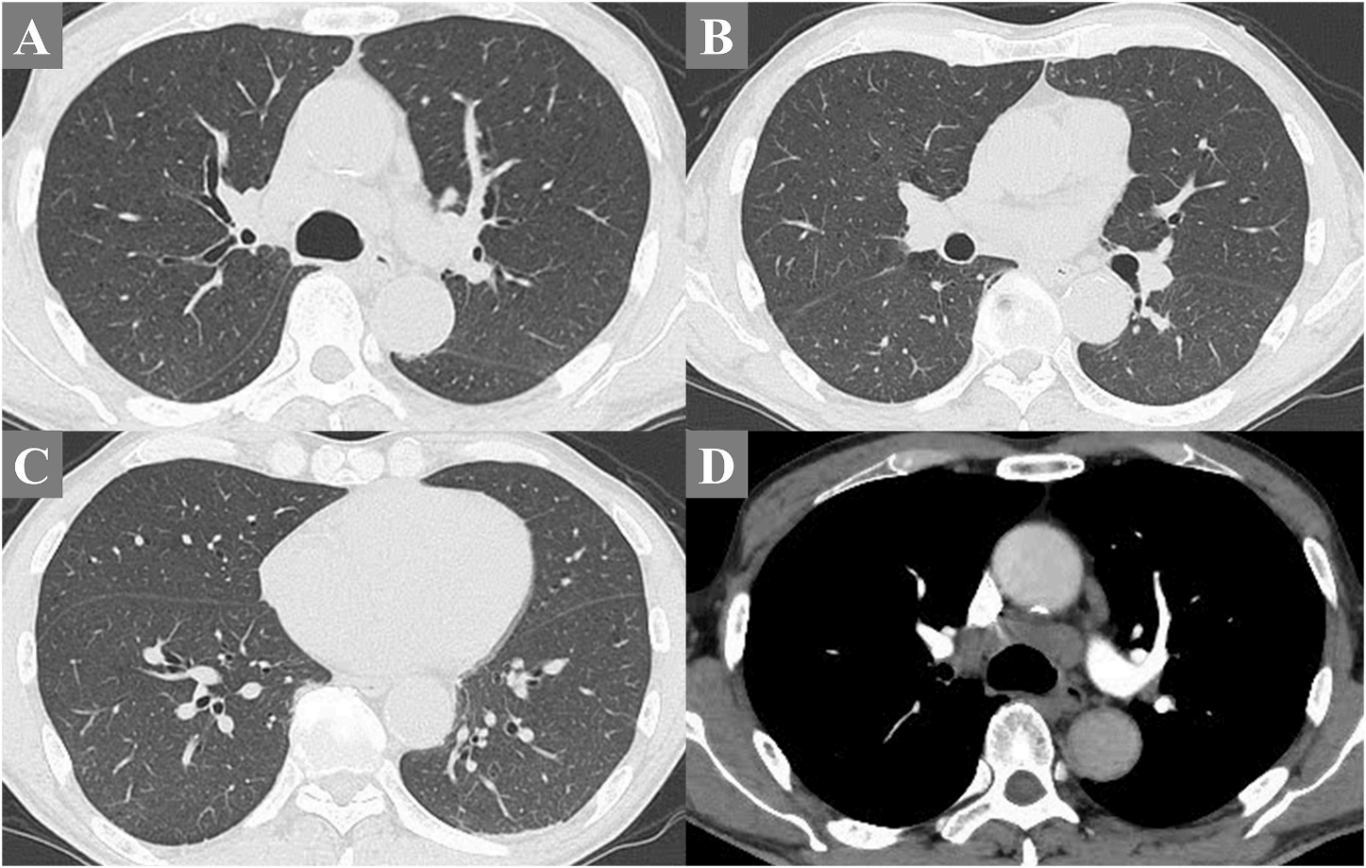
In the plain chest computed tomography (CT), minimal, diffuse increase in pulmonary field density can be observed, with almost normal appearance (A–C). Contrast-enhanced chest CT demonstrates enlargement of the mediastinal hilar lymph nodes (D).

At the time of the emergency department visit on admission, he was alert but had reduced vitality. His vital signs were as follows: blood pressure, 140/78 mmHg; temperature, 36.0 °C; heart rate, 78 bpm; respiratory rate, 16 breaths/min, and oxygen saturation, 88 % at room air and 98 % on 2 L/min via nasal cannula. Physical examination revealed clear bilateral breathing sounds with no asymmetry, cervical lymphadenopathy, or rashes. Complete blood counts showed a white blood cell count of 3700/μL and hemoglobin level of 12.1 g/dL. The serum tests revealed lactate dehydrogenase level (LDH) of 506 U/L, C-reactive protein (CRP) level of 1.34 mg/dL, sodium level of 125 mEq/L, and absence of anti-nuclear and anti-neutrophil cytoplasmic antibodies (Table 1). Arterial blood gas analysis revealed a pH of 7.438 mmHg, partial pressure of arterial carbon dioxide of 31.7 mmHg, and partial pressure of arterial oxygen of 95.0 mmHg, while receiving 2 L/min of nasal oxygen. Chest X ray showed no remarkable findings (Fig. 1). Chest CT showed minimal diffuse ground-glass opacities and mild bilateral mediastinal lymphadenopathy (Fig. 2). In addition, contrast-enhanced CT of the chest and lower extremities did not reveal any obvious thrombus. The hormonal secretion disorders were considered to be due to possible panhypopituitarism based on the low levels of TSH and cortisol (Table 1), and oral treatment with 15 mg of cortisol and 50 μg of levothyroxine was initiated. Transbronchial needle aspiration (TBNA) was performed on the mediastinal lymph node #4R; however, no clear malignant findings were observed at this time point. Pulmonary ventilation scintigraphy showed no apparent defects. Respiratory function tests indicated a low diffusion capacity for carbon monoxide of 54 %. Although transthoracic echocardiography revealed no abnormalities in left ventricular contractility or dominant valvular lesions, a mild elevation of the tricuspid regurgitation pressure gradient to 24 mmHg was noted. However, right heart catheterization revealed pulmonary artery pressure of 30/14/21 mmHg, ruling out significant pulmonary hypertension, and no evidence of shunting was observed. With continued treatment for idiopathic adrenal insufficiency, the dyspnea gradually improved. Consequently, the patient requested discharge for outpatient follow-up. On the 42nd day of hospitalization, home oxygen therapy (HOT) was initiated, and the patient was discharged.

**Table 1:** Laboratory findings on admission.

Hematology			Biochemistry			Serology		
WBC	3700	/μL	TP	6.8	g/dL	CEA	2.1	ng/mL
Neu	43.0	%	Alb	3.1	g/dL	NSE	21.2	ng/mL
Lym	26.9	%	T-Bil	0.6	mg/dL	CYFRA	1.5	ng/mL
Mono	19.8	%	AST	36	U/L	ProGRP	73.5	pg/mL
Eos	7.9	%	ALT	21	U/L	BNP	23.6	pg/mL
Baso	0.0	%	LDH	506	U/L	sIL-2R	1518	U/mL
RBC	396	10^4^/μL	ALP	87	U/L			
Hb	12.1	g/dL	γ-GTP	18	U/L	**Endocrine Tests** (15 days before admission)		
Hct	33.6	%	BUN	16	mg/dL			
Plt	21.8	10^4^/μL	Cre	0.85	mg/dL	TSH	0.054	μU/mL
			CK	40	U/L	FT4	0.72	ng/dL
			CRP	1.34	mg/dL	cortisol	4.9	μg/dL
**Coagulation**			Na	125	mEq/L	ACTH	10.1	pg/mL
PT-INR	1.17		K	3.9	mEq/L			
APTT	36.1	sec	Cl	93	mEq/L			
			Glu	88	mg/dL			
			HbA1c	6.0	%			

WBC, white blood cells; Neu, neutrophils; Lym, lymphocytes; Mono, monocytes; Eos, eosinophils; Baso, basophils; RBC, red blood cells; Hb, hemoglobin; Hct, hematocrit; Plt, platelets; PT-INR, prothrombin time-international normalized ratio; APTT, activated partial thromboplastin time; TP, total protein; Alb, Albumin; T-Bil, total-bilirubin; AST, aspartate transaminase; ALT, alanine transaminase; LDH, lactate dehydrogenase; ALP, alkaline phosphatase; γ-GTP, γ-glutamyltransferase; BUN, blood urea nitrogen; Cre, creatine; CK, creatine kinase; CRP, C-reactive protein; Glu, glucose; HbA1c, glycosylated hemoglobin A1c; CEA, carcinoembryonic antigen; NSE, neuron-specific enolase; CYFRA, cytokeratin fragment; ProGRP, progastrin-releasing peptide; BNP, brain natriuretic peptide; sIL-2R, soluble interleukin-2 receptor; TSH, thyroid stimulating hormone; FT4, free-T4; ACTH, adrenocorticotropic hormone.

The patient was initially scheduled for further outpatient investigation of respiratory failure; however, 4 days after discharge, he contracted coronavirus disease (COVID-19) and was readmitted. Given the associated oxygen requirement, the patient was classified as having moderate-severity grade II COVID-19, and treatment with steroids and remdesivir was initiated. Ten days after COVID-19 treatment, the respiratory failure improved, and oxygen saturation was maintained without the need for HOT. The patient was discharged home on the 15th day of hospitalization.

**Fig. 3:**
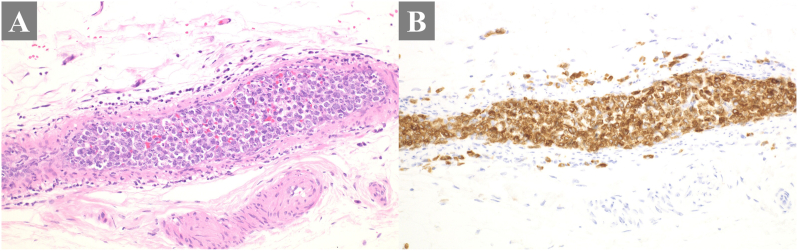
Histopathology of the organ biopsy specimens. There were atypical lymphoma cells filled into the capillary vessels (A) (Hematoxylin and Eosin staining: × 200 magnification). The neoplastic cells were positive for CD79a (B) (Immunohistochemical staining for CD79a: × 200 magnification).

**Fig. 4:**
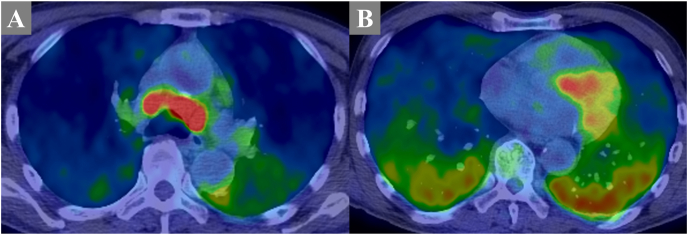
In the positron emission tomography (PET)/computed tomography (CT), abnormal uptake of fluorodeoxyglucose was detected in mediastinal lymph nodes and bilateral lungs (A, B).

However, 3 weeks later, the patient presented to the outpatient clinic with worsening dyspnea, leading to admission on the same day. On admission, the patient required 5 L/min of oxygen. Video-assisted thoracoscopic surgery was considered to investigate the cause of the respiratory failure; however, owing to the severity of the respiratory failure, the surgical risk was deemed high. Various imaging and respiratory function tests were conducted to investigate the cause of respiratory failure. Given the nonspecific findings, improvement in respiratory failure with steroid use during COVID-19 treatment, and high level of LDH, IVLBCL, a malignant lymphoma, was considered in the differential diagnosis. On the 8th day, random skin biopsies were performed on the left abdominal inside, left abdominal outside, and right thigh. Pathological examination revealed large, atypical lymphocytes filling the vessels within the adipose tissue (Fig. 3). Immunostaining was positive for CD79 and PAX5, leading to a diagnosis of IVLBCL. Positron emission tomography revealed enlarged cervical and supraclavicular lymph nodes on the right side, and increased ^18^F-fluorodeoxyglucose uptake in the pituitary gland and bilateral posterior lungs (Fig. 4). Therefore, the clinical presentation initially thought to be idiopathic adrenal insufficiency was reconsidered as an indication for IVLBCL. Upon reviewing the TBNA specimen from bronchoscopy, cells presumed to be enlarged lymphocytes were found to be diffusely positive for CD79a and predominantly positive for PAX-5. Based on the immunostaining results, the tissue from the mediastinal lymph node showed findings consistent with IVLBCL, similar to those observed in random skin biopsy. A bone marrow biopsy did not show any evidence of IVLBCL.

Thus, based on these findings, 4 months after the initial visit, treatment with rituximab, cyclophosphamide, vincristine, doxorubicin, and prednisolone was initiated promptly. By the fourth day of chemotherapy, respiratory failure had improved, and oxygen supplementation was no longer required. After two courses of chemotherapy for IVLBCL, the baseline values of TSH, cortisol, and adrenocorticotropic hormone showed an increasing trend. Based on these results, IVLBCL was considered the cause of panhypopituitarism based on the endocrinologist's assessment. However, supplementation therapy with corticosteroids and levothyroxine was continued because the adrenal function had not fully normalized. Twelve months after the initial visit, there was no worsening of respiratory failure, and the patient continues outpatient visits for IVLBCL and chemotherapy.

## 3 Discussion

In our patient, despite exhaustive investigation of type 1 respiratory failure, hypoxemia, and mild pulmonary hypertension, a definitive diagnosis in the early stages of disease onset was elusive. The onset of COVID-19 infection that led to treatment initiation was a turning point, narrowing the differential diagnosis to a condition responsive to corticosteroids. A high level of LDH was also a clue for the differential diagnosis of IVLBCL [11]. Subsequently, through a random skin biopsy, we obtained a pathological diagnosis of IVLBCL. Finally, chemotherapy improved the respiratory failure. The clinical course and diagnostic process of this case provide valuable insights for clinicians.

IVLBCL generally occurs with lung involvement; however, cases in which lung lesions precede other manifestations of IVLBCL are rare [12]. Moreover, the symptoms and test results of pulmonary involvement in IVLBCL are often nonspecific, making early differentiation challenging [4]. This was a rare case of IVLBCL with primary lung involvement. Additionally, the multitude of nonspecific symptoms makes it difficult to consider IVLBCL as a differential diagnosis. Consequently, treatment initiation occurred 7 months after symptom onset and 3 months after seeking medical attention, reflecting a prolonged period between diagnosis and treatment initiation. By the time the treatment commenced, severe respiratory failure necessitating 5 L of oxygen had developed. Therefore, it is reasonable to consider that any further delay in treatment initiation might have resulted in a potentially fatal outcome. IVLBCL should be considered in the differential diagnosis when unexplained hypoxemia and elevated LDH levels are encountered.

The diagnosis of IVLBCL requires histological evidence of tumor cell proliferation within the blood vessels [13]. In 1999, Kraus et al. histologically identified IVLBCL in the skin tissue that appeared grossly normal during autopsy [13]. Since then, biopsy of clinically normal-appearing skin has been established as a useful diagnostic method and is now widely performed alongside bone marrow examination in the differential diagnosis of IVLBCL [[11], [14], [15]]. In the present case, severe respiratory failure requiring 5 L of oxygen made lung biopsy challenging. Therefore, a random skin biopsy yielded conclusive evidence for a diagnosis. In cases of IVLBCL, even when presenting with respiratory failure, initiating chemotherapy is crucial, as it can lead to improvements in both the respiratory status and overall condition [11].

## 4 Conclusion

In conclusion, we encountered a case of IVLBCL diagnosed through random skin biopsy in a patient presenting with unexplained respiratory failure. When encountering cases of unexplained hypoxemia and elevated LDH levels, considering IVLBCL in the differential diagnosis and planning a random skin biopsy are deemed advisable.

## Funding

None.

## CRediT authorship contribution statement

**Haruka Fujioka:** Data curation, Visualization, Writing – original draft. **Kei Nakashima:** Supervision, Writing – review & editing. **Yuka Uesugi:** Writing – review & editing. **Miki Nyui:** Data curation, Writing – review & editing. **Naoki Inoshima:** Writing – review & editing. **Jun Hayashi:** Writing – review & editing. **Michinori Yoshimi:** Data curation, Writing – review & editing. **Miho Katsumata:** Data curation, Writing – review & editing. **Masahiro Masuzawa:** Supervision, Writing – review & editing. **Hayato Usami:** Data curation, Writing – review & editing. **Akane Ito:** Data curation, Writing – review & editing. **Rikako Tabata:** Data curation, Writing – review & editing. **Kosei Matsue:** Supervision, Writing – review & editing.

## Declaration of competing interest

The authors declare that they have no known competing financial interests or personal relationships that could have appeared to influence the work reported in this paper.
